# The right to deworming: The case for girls and women of reproductive age

**DOI:** 10.1371/journal.pntd.0006740

**Published:** 2018-11-21

**Authors:** Theresa W. Gyorkos, Antonio Montresor, Vicente Belizario, Beverley-Ann Biggs, Mark Bradley, Simon J. Brooker, Martin Casapia, Philip Cooper, Sila Deb, Nicolas L. Gilbert, Rubina Imtiaz, Virak Khieu, Stefanie Knopp, Ornella Lincetto, Layla S. Mofid, Denise Mupfasoni, Cori Vail, Jozef Vercruysse

**Affiliations:** 1 World Health Organization Collaborating Centre for Research and Training in Parasite Epidemiology and Control, Department of Epidemiology, Biostatistics and Occupational Health, McGill University, Montreal, Quebec, Canada; 2 Department of Control of Neglected Tropical Diseases, World Health Organization, Geneva, Switzerland; 3 Department of Parasitology, College of Public Health, University of the Philippines-Manila, Philippines; 4 Department of Medicine, University of Melbourne, Parkville, Victoria, Australia; 5 GlaxoSmithKline, Twickenham, United Kingdom; 6 Neglected Tropical Diseases-Global Health, Bill and Melinda Gates Foundation, Seattle, Washington, United States of America; 7 Ciencias Médicas y de la Salud, Universidad Nacional de la Amazonia Peruana and Asociación Civil Selva Amazónica, Iquitos, Peru; 8 Department of Epidemiology of Infectious Diseases, St. George’s, University of London, London, United Kingdom; 9 Child Health, Ministry of Health and Family Welfare, New Delhi, India; 10 World Health Organization Collaborating Centre for Research and Training in Parasite Epidemiology and Control, Department of Epidemiology, Biostatistics and Occupational Health, Montreal, Quebec, Canada; 11 Children Without Worms, Atlanta, Georgia, United States of America; 12 National Helminth Control Programme, National Centre for Parasitology, Entomology and Malaria Control, Ministry of Health, Phnom Penh, Cambodia; 13 Department of Epidemiology and Public Health, Swiss Tropical and Public Health Institution and University of Basel, Basel, Switzerland; 14 Family, Women’s and Children’s Health, Maternal, Newborn, Child and Adolescent Health, Policy, Planning and Programmes, World Health Organization, Geneva, Switzerland; 15 STH Global Public Health, Johnson and Johnson, Raritan, New Jersey, United States of America; 16 Department of Virology, Parasitology, and Immunology, Ghent University, Merelbeke, Belgium; Brown University, UNITED STATES

Girls and women of reproductive age (WRA) bear a large burden of disease from soil-transmitted helminth (STH) infections worldwide. This burden is primarily attributable to the anemia caused by hookworm and *Trichuris trichiura* infections [1,2]. Together with preschool children (pre-SAC) and school-age children (SAC), WRA are one of the three risk groups most vulnerable to STH morbidity [3,4,5,6], yet the benefits and opportunities for addressing STH among this group have, until recently, been under-appreciated.

Mupfasoni and colleagues estimate that over 688,000,000 WRA living in STH-endemic countries were in need of deworming treatment in 2015 [5], and Montresor and colleagues have further estimated that more than 600,000 disability-adjusted life years (DALYs) are lost by WRA due to STH, annually [7]. Although coverage rates of deworming in the two child risk groups have been steadily increasing (from less than 10% in 2003 to more than 60% in 2016 [8]) through school or child health campaign platforms, coverage in WRA has not improved. Data on treatment coverage for WRA are not routinely collected by national programs, but it is likely that, as countries successfully transition out of lymphatic filariasis treatment programs (in which albendazole is included in the treatment regimen), coverage rates of deworming in WRA may decline. Moreover, although 2002 World Health Organization (WHO) guidance [9] recommended pregnant women should be treated, uptake of treatment during antenatal care has been low, due (in part) to a perceived fear of side effects, especially teratogenicity, among women and health personnel. Operational research is currently underway to identify an efficient approach to ruling out first-trimester pregnancy to respond to these concerns [10]. In order to expand treatment among WRA, new community-based strategies are required, with strategies tailored to reaching each of the different subgroups of WRA (i.e., adolescent girls, pregnant women, lactating women, and nonpregnant nonlactating women) [11,12]. For example, deworming can be added to iron supplementation programs targeting pregnant women, and it can be offered to lactating women at well-baby clinics.

Several government-supported platforms already exist in many countries to reach adolescent girls (e.g., schools), pregnant women (e.g., antenatal care clinics), and lactating women (e.g., postpartum and well-baby clinics). In addition, women can be reached by piggybacking onto other highly successful campaigns like Child Health Days and new efforts to increase universal health care coverage [13,14]. There are also many untapped opportunities to reach the majority of at-risk WRA and innovative approaches (e.g., use of social technologies, among others) will be needed to ensure that coverage is optimal. With these opportunities also come challenges that will need to be overcome. First, there is no drug donation program for WRA, with current donation programs for SAC only. Second, special considerations need to be made for women who are pregnant, as deworming is contraindicated in the first trimester. Third, appropriate messaging is required to educate women and health personnel of the safety and benefits of deworming in order to help allay fears of perceived side effects. Finally, there are a number of outstanding basic and operational research issues that merit further investigation, including monitoring and evaluating the impact of deworming programs targeting WRA on maternal and infant outcomes and evaluating the cost-effectiveness of different platforms for reaching WRA, among others.

**Fig 1:**
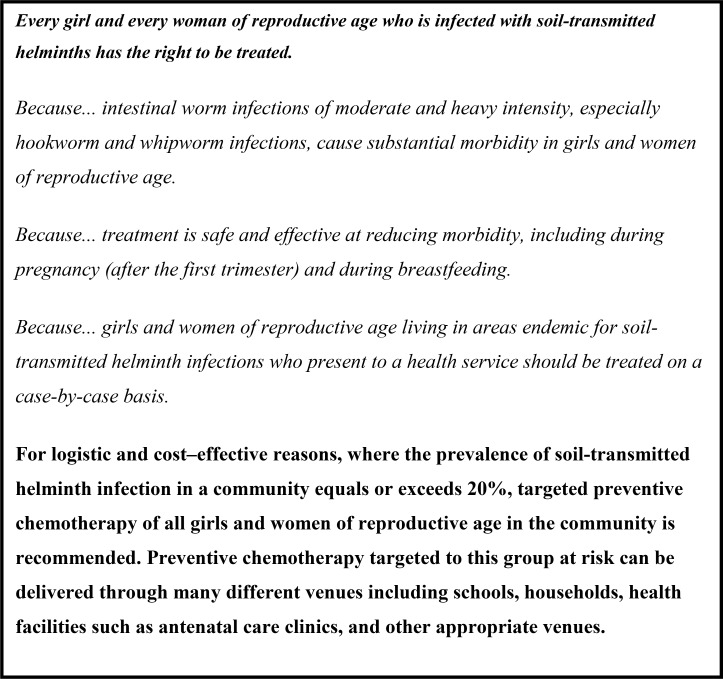
The Bellagio Declaration.

Clear policy and guidance are also essential to support country efforts to expand routine deworming of WRA, and two recent WHO publications have provided the necessary policy framework. The first is the new evidence-informed guideline on preventive chemotherapy which documents deworming recommendations for all three high-risk groups (SAC, pre-SAC, and WRA [including adolescent girls]) [15]. It reaffirms deworming in all at-risk WRA subgroups, including pregnant women, after the first trimester. The second, a report of an international expert advisory group meeting, is the 2018 update to the 1994 Report of the WHO Informal Consultation on Hookworm Infection and Anemia in Girls and Women [16, 17]. This report, focusing specifically on WRA, summarizes the group's deliberations, outlining a set of recommendations and research priorities, and informing the meeting's unanimously adopted Bellagio Declaration (Fig 1).

This declaration represents a seminal landmark for the promotion of the rights and livelihoods of WRA living in STH-endemic areas of the world. All stakeholders in women's health should take immediate action in their respective domains to ensure that WRA are now included in their STH policies, programs, and clinical and operational research priorities. Such collective action will ensure that every girl and woman of reproductive age at-risk of STH can benefit from effective treatment.
